# Insomnia and circadian misalignment: an underexplored interaction towards cardiometabolic risk

**DOI:** 10.5935/1984-0063.20200025

**Published:** 2021

**Authors:** Barbara Nobre, Isabel Rocha, Charles M. Morin, Miguel Meira e Cruz

**Affiliations:** 1 Centro Cardiovascular da Universidade de Lisboa, Lisbon School of Medicine, Sleep Unit - Lisbon - Portugal.; 2 Centro Cardiovascular da Universidade de Lisboa, Lisbon School of Medicine, Cardiovascular Autonomic Function Lab - Lisbon - Portugal.; 3 Laval University, Psychology - Québec - Québec - Canada.; 4 Faculdade São Leopoldo Mandic, Laboratory of Neuroimmune Interface of Pain Research - Campinas - Sao Paulo - Brazil.

**Keywords:** Insomnia, Circadian Misalignment, Cardiometabolic Risk, Shift-Work, Autonomic Nervous System

## Abstract

Insomnia remains the most prevalent sleep disorder worldwide, and its pathophysiology suggests an interface with circadian rhythm sleep-wake disorders (CRSWDs). Some epidemiological studies have linked insomnia and circadian misalignment with adverse cardiometabolic outcomes, but the mechanisms underlying this relationship are still unclear. The autonomic nervous system (ANS) has been pointed out as a crucial/key mediator that triggers cardiometabolic risk. Therefore, a critical review of the literature focused on the past ten years was conducted to highlight the relationship between insomnia, circadian misalignment and cardiometabolic risk, with particular emphasis on the inﬂuence of the ANS. Shift work, as a model of circadian misalignment, was shown to increase both cardiovascular and metabolic risk and so may integrate a proof of concept on this link. Furthermore, there is good evidence from previous studies supporting that cardiac autonomic dysfunction is indeed a possible mechanism that potentiates cardiometabolic risk in insomniacs and individuals with a misalignment of the circadian timing system (e.g., shift workers), via changes in autonomic variables. Further research is however required in order to deﬁnitively establish this interactive relationship.

## INTRODUCTION

Insomnia is a highly prevalent sleep disorder affecting 7% of the European and 9% to 20% of the American adult population^1^ with devastating effects on psychological^2,3^ and cardiometabolic health^4^. As insomnia has great health-related^5^ and economic related impact^6,7^, effective therapeutic options were developed through the cognitive-behavioral^8^ and pharmacological domains^9^. However, the relapse rates are high^10^ and the field has struggled to develop preventive measures^1^ acting on specific and easily identified stressors with well-known interaction with shortened and disturbed sleep^11^.

The circadian timing system is a complex endogenous machinery regulating nearly all physiologic functions including sleep/wakefulness^12^. Hence, sleep/wake cycles are vulnerable to circadian disruption^13^, which is therefore a risk factor for the development of circadian rhythm sleep-wake disorders (CRSWDs). These disorders are characterized by misalignment of the circadian clock regarding the environmental cycle, which tends to result in sleep deprivation, excessive sleepiness during wake hours, and insomnia symptoms^14^. Among sleep disorders are chronic insomnia associated with an altered endogenous circadian clock, i.e., that could run slower or faster than the norm^12,14^. CRSWDs and insomnia often occur in combination.

The potential health implications of insomnia are well known, namely in psychological issues (e.g., anxiety, depression and stress)^15,16^ and cardiovascular disease (e.g., hypertension, heart failure, coronary heart disease, etc.)^17^. Similarly, the effects of shift work exacerbated by circadian misalignment on cardiometabolic risk have been demonstrated^18^. However, the metabolic consequences and mechanisms involved in these relationships are still misunderstood^13,19^.

**Figure 1:**
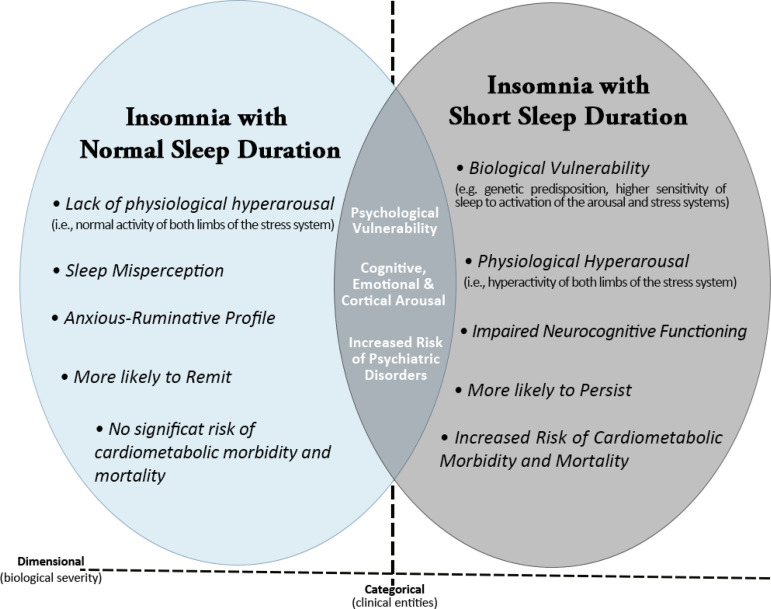
Heuristic model of the underlying pathophysiological mechanisms and clinical characteristics of the two insomnia phenotypes based on objective sleep duration. The common characteristics of the two phenotypes are presented in the overlapping area, while their unique characteristics are presented in the areas of each phenotype that do not overlap. (Adapted from Vgontzas et al.^25^).

**Figure 2:**
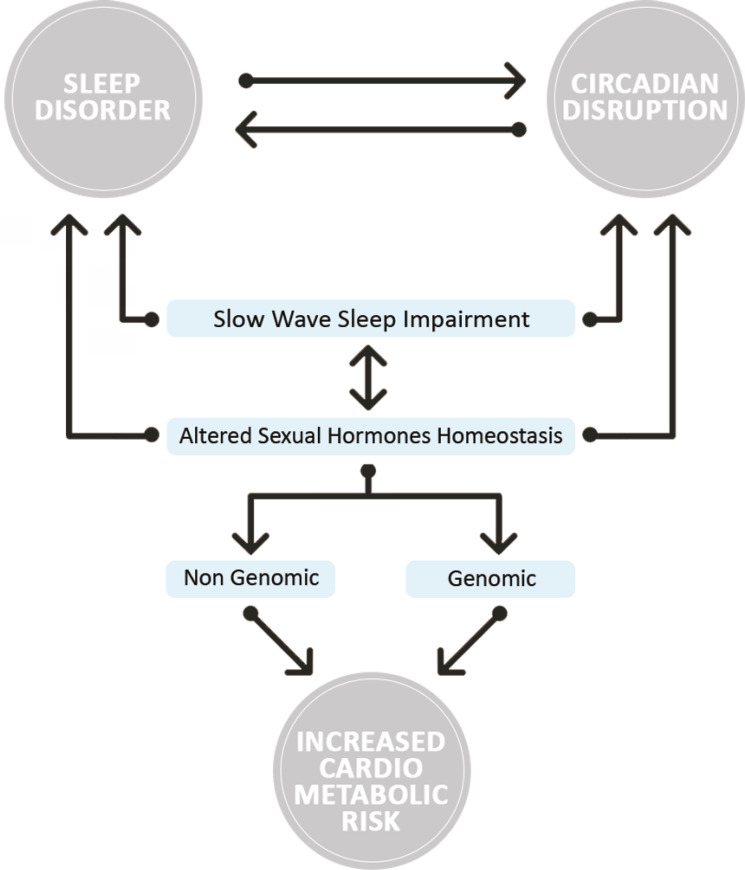
Illustration on the dynamics of androgenic hormone secretion as an intermediate mediator in the link between slow-wave sleep loss and cardiometabolic risk. (Adapted from Meira e Cruz and Gozal^55^).

**Figure 3:**
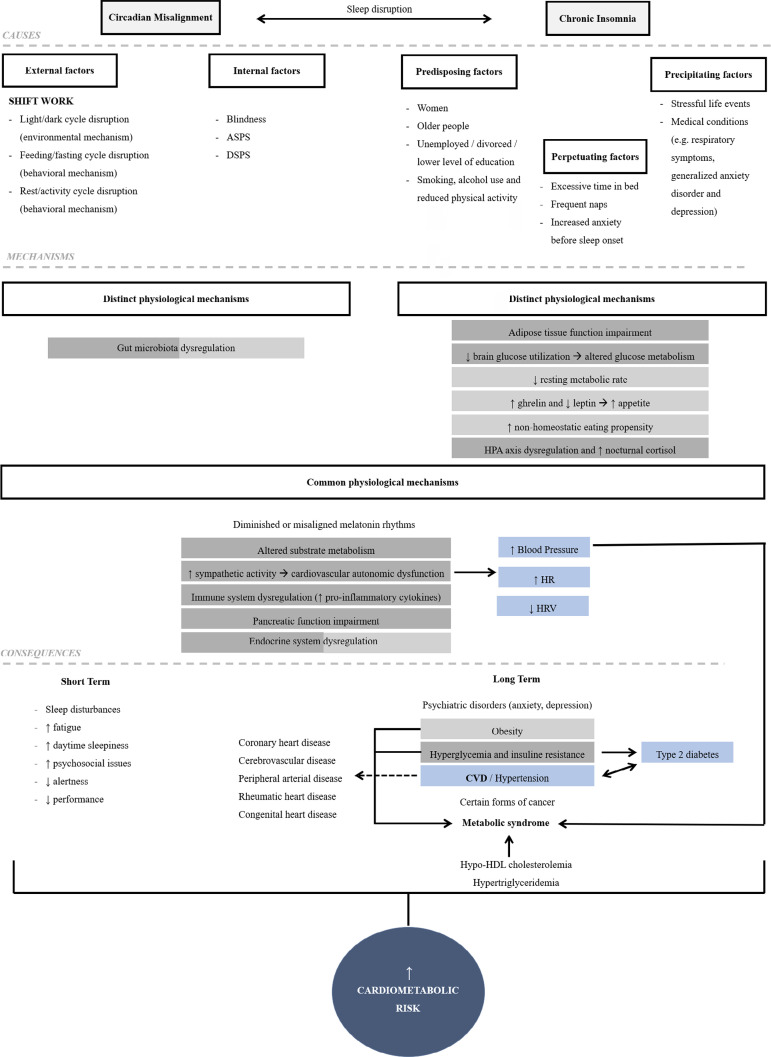
Schematic representation of the risk factors, possible pathophysiological pathways (in common and distinct) linking circadian misalignment and insomnia to short term and long-term health consequences. Inconsistencies in the literature and future investigations may bring some differences regarding the mechanisms listed as distinct, showing that some of them may also occur in both disturbances. The colors represent the link between cause and effect, i.e., the mechanisms represented in green contribute to the development of the consequence in green (hyperglycemia and insulin resistance). The same is true for the mechanisms in orange (which result in obesity) and blue (CVD, hypertension and diabetes). ↑: increased; ↓: reduced; ASPS: Advanced Sleep Phase Syndrome; DSPS: Delayed Sleep Phase Syndrome; HPA: Hypothalamic-Pituitary-Adrenal Axis; HR: Heart Rate; HRV: Heart Rate Variability; CVD: Cardiovascular Disease; HDL: High-Density Lipoprotein.

We briefly describe insomnia, their risk factors and epidemiology. A review of the literature on the causal link between insomnia and cardiometabolic risk and between circadian timing, circadian misalignment, and its relevance to shift work will follow, extending to the role of circadian rhythms in the genesis of insomnia and their impact on cardiometabolic function as illustrated in Figures 1 to 3. To further elucidate the exact mechanisms explaining these interactive relationships we have pursued to also review the studies published in the last decade dedicated to the ANS.

## INSOMNIA

Sleep, which is affected by lifestyle and health, is a restorative process and has a major influence on protein synthesis and hormone release^20^. Adequate duration and quality of sleep improve alertness, mood and performance, besides long-term health benefits^21^. We can easily understand its importance by the fact we spend a third of our time sleeping and the productivity of the other two-thirds depends on the quality of sleep we have^22^.

Insomnia is the most reported sleep problem in industrialized countries worldwide and somehow it can be characterized as a state of cerebral hyperexcitability or hyperarousal^16,23^. Hyperarousal results from an elevated whole-body metabolic rate during sleep and wakefulness, increased cortisol secretion during the early sleep period, and reduced parasympathetic activity in heart rate variability^24^. According to the International Classification of Sleep Disorders (ICSD) and the fifth edition of the Diagnostic and Statistical Manual of Mental Disorders (DSM-5, 2013), insomnia is defined as a difficulty of falling asleep (onset), staying asleep (maintenance), early awakening, and associated daytime functioning complaints^4,6,25^. Despite the higher prevalence of mixed symptom phenotypes, sleep-onset insomnia is more common in younger adults, and sleep-maintenance difficulties are more frequent in middle-aged and older adults. People with this problem must be dissatisfied with their sleep and experience one or more of the following symptoms: fatigue, decreased energy, difficulty concentrating, mood disturbances and decreased performance at work or school. These are required criteria to make the diagnosis of insomnia disorder^2^.

Note that there is an important distinction between insomnia symptoms, that typically last a few days or weeks, and insomnia disorder, which tends to be persistent and often lasts months or years. The diagnosis of chronic insomnia requires sleep difficulties for ≥3 nights per week and last for >3 months^2,26^. Among the general population, 30-40% suffers from insomnia symptoms and 10-15% from chronic insomnia, i.e., as a sleep disorder of its own^2,4^. People with insomnia disorder may benefit from some form of treatment to help them get back to healthy sleep patterns. This condition is commonly linked to medical or psychiatric issues such as anxiety, depression and burnout, although sometimes it is difficult to understand this cause-and-effect relationship, and its mechanistic pathways^15,16,26,27^. A recent report about the incidence per annum of acute insomnia showed that this rate is indeed remarkably high, but the majority incident cases resolve within a few days to weeks. On the other hand, incident chronic insomnia only occurs in about 2 in 100 individuals^28^. Current prevalence of insomnia affects about 7% to 20% across studies^1^.

Despite the heterogeneity of the disorder, the three stages of insomnia: acute, early and chronic are influenced, to different degrees, by various factors. This risk factors include: 1) predisposing factors, which contributes to the development of the disorder (demographic, biologic, psychological and social characteristics); 2) precipitating factors, which are the real trigger of an acute episode of insomnia (stressful life events or medical conditions that may disrupt sleep); and 3) perpetuating factors, which potentiate sleep disturbances even after the initial trigger has been removed (behavioral or cognitive changes like excessive worrying about sleep loss and its effects). In chronic insomnia, the perpetuating factors have a stronger contribution to the maintenance than the onset of the disorder^2,24^. In summary, some risk factors that influence insomnia include increased age, female sex, comorbid disorder (medical, psychiatric, sleep and substance use), shift work, unemployment/lower socioeconomic status, a positive family history of insomnia^2,26^ and higher scores on the FIRST, the Ford Insomnia Response to Stress Test (see Drake et al.^29^ for the whole instrument)^1,30^.

Since insomniacs often experience stressful life events, stress and psychosocial factors are closely connected with the pathogenesis of this disorder, which subsides on a hyperarousal model^31^. It is expected that insomnia activate the stress system, specifically the Hypothalamic-Pituitary-Adrenal axis (HPA), and sympathetic system. The prolonged activation of both systems causes increased arousal and sleeplessness^31,32^. Under stressful conditions, there is a dysregulation of the HPA-axis with changes in the circadian rhythmicity of cortisol. Ultradian cortisol pulses are believed to be involved in the maintenance of wakefulness during the day and their absence at night allows the consolidation of sleep and/or shorter nighttime awakenings^33^.

Regarding the standard tool in sleep medicine for evaluating sleep-related pathophysiology, the polysomnography (PSG), two different phenotypes of the disorder have been proposed: insomnia with objective near-normal sleep duration (PSG-defined total sleep time (TST) ≥6h) and insomnia with objective short sleep duration (PSG- defined TST<6h), the latter is expected to be a more severe biological phenotype of this sleep disorder^25,34^. For instance, a prospective study called Sleep Heart Health Study showed that 48% of the 631 participants also had a sleep duration of <6h on PSG, beyond insomnia symptoms^17^. Figure 1 displays the common and distinct effects of insomnia with these two phenotypes on mental and physical health.

### Insomnia and cardiometabolic risk

Given the importance of good sleep, in either quantity or quality, it is not surprising that sleep disturbances may be a risk factor for medical conditions, contributing to the development of adverse long-term health outcomes. Cardiometabolic risk can be defined as a cluster of metabolic and cardiovascular abnormalities, such as obesity, insulin resistance, hypertension and atherosclerosis. These risk factors predispose individuals to cardiovascular disease (CVD) and type 2 diabetes^33,35^. Therefore, understanding the causes, factors, and mechanisms that perpetuate insomnia is considered a major public concern. See Figures 2 and 3 for more detailed and summarized information.

A wide range of evidence supports that both acute and chronic insomnia have been associated with adverse long-term health consequences, such as diabetes, hypertension and CVD^4,15,23,25,27,36-38^, overall contributing to a worse quality of life of the individuals. Some mechanisms underlying the relationship between insomnia and CVD comprise the dysregulation of the HPA axis, abnormal modulation of the autonomic nervous system (ANS) with a global sympathetic overactivity and increased systemic inflammation^17^. However, the metabolic consequences of insomnia are still unclear^13^.

Because of the variation in how insomnia is defined and measured, there are conflicting data and some inconsistencies in the literature. Until 2013, the connection between insomnia severity and/or short sleep duration and medical morbidity was not well established, leading some sleep researchers to study this relationship in more detail^25^.

In the last decade, several observational studies have demonstrated that CVD remains the leading cause of mortality for both men and women worldwide^39^, with an estimated prevalence rate of 30% to 35%^40^. Together with the fact that insomnia might be associated with the development of CVD morbidity and mortality, providing an overall increased relative risk ranging from 1.2- to 3.9-fold for CVD^17,40^, it would be logical to elucidate how insomnia might lead to potentially life-threatening cardiovascular and metabolic diseases. We will try to summarize the data available from previous studies and highlight the magnitude of this relationship, particularly if insomnia accompanied by short sleep duration confers a higher risk of major cardiometabolic events.

Recent studies report that patients with insomnia with objective short sleep duration have a higher risk of cardiovascular risk factors^25^, poor treatment response (especially to non-pharmacological therapies), and illness recurrence^26,36^, due to multiple mechanisms underlying this relationship (Figure 1). Therefore, it has been suggested that insomnia with objective short sleep time might be a unique phenotype of insomnia disorder that negatively affects the variability of blood pressure and heart rate and is associated with hypertension^38^, type 2 diabetes^37^ and CVD risk, because of the dysregulation of the HPA^4,25^, which also contributes to the activation of both limbs of the stress system^36^. Patients with this insomnia phenotype usually have impaired glucose and lipid metabolism^41^, insulin resistance^4^, loss of pancreatic β-cell function, increased inflammation (higher levels of pro-inflammatory biomarkers such as IL-6, TNF and CRP^4^), increased cortisol levels, increased food intake, weight gain and obesity^37^, besides alterations in cardiovascular autonomic control, such as increased sympathetic activity and neurocognitive-physiologic arousal^4,38,42-44^ (Figure 3). Walsh^39^ suggested that future investigations of sleep-related CVD risk should consider insomnia symptoms and sleep duration as considering one feature may provide an incomplete characterization of clinically relevant sleep phenotypes and their impact on health outcomes. Since healthy people experience a 10-20% decrease in blood pressure (BP) at night, those who do not exhibit this “dip” of at least 10% change in resting BP are called “non-dippers”^45^. In this regard, some studies have suggested a link between BP non-dipping and insomnia, i.e., observational studies reporting that there are more non- dippers of blood pressure among subjects with chronic insomnia relative to good sleepers^38,46-49^. Hence, non-dipping BP is associated with higher risk for hypertension and it could be one mechanism linking insomnia and cardiovascular morbidity and mortality^46^.

However, some results focused on the connection of insomnia with PSG-short sleep to cardiometabolic risk factors are mixed. For instance, the results of D’Aurea et al.^36^ showed that shorter sleep was not associated with differences in body mass index (BMI) and body composition, and Leblanc et al.^37^ revealed that although sleep loss seems to increase the risk of developing diabetes via multiple pathways as we mentioned before, the specific causal mechanisms were undetermined. A third study, Whitesell et al.^20^, reported that insomnia is correlated with hypertension, but a causal relationship has not been established and according to Tobaldini et al.^4^, whether the relationship between short sleep duration and cardiometabolic disorders is monodirectional or bidirectional is still debated. Since all evidence is correlational/observational, not experimental, we must be cautious in interpreting the data.

### Circadian misalignment: from pathophysiology to clinical implication

The *circadian* (lit. “about a day”)^50^ timing system aligns oscillations in biological processes such as food intake, sleep-wake cycles, both systolic and diastolic blood pressure^45^, and energy expenditure to the earth’s solar day, producing rhythms in physiology and behavior^51^. These rhythms are controlled by the central circadian pacemaker in the suprachiasmatic nucleus (SCN) of the hypothalamus and they help to synchronize molecular circadian clocks in peripheral cells and tissues, including the liver muscle, adipose tissue and pancreas^52-54^. The coordination between behavioral responses (i.e., sleep-wake, feeding-fasting), metabolic responses (lipid and glucose metabolism), and blood pressure with the light/dark cycle involves the autonomic innervation and/or endocrine signals^43,50,55^. This is extremely important to the species because it allows them to anticipate and adapt to the 24h day/night cycle^4,25^.

Therefore, when the endogenous circadian rhythms are not in synchrony with either the environment or each other, as a result of inadequate meal timing and/or sleep and wakefulness sleep misalignment in relation to other rhythms, circadian disruption occurs. A mismatch of circadian rhythms triggers a cascade of biological changes that have potential effects on brain-body connections and influence the human homeostatic systems^42,56^ (see Figure 2).

If the desynchronization between internal sleep-wake rhythms and light-dark cycles is maintained, circadian rhythm sleep-wake disorders (CRSWDs) may arise in the form of persistent: 1) delayed sleep phase syndrome (DSPS); 2) advanced sleep phase syndrome (ASPS); and, 3) irregular sleep-wake rhythm (ISWR); periodic: free-running disorder (non-24-hour sleep-wake disorder), mostly seen in blind people; or transient, as a result of the external environmental and/or social circumstances (shift work and jet lag syndrome)^12,14^.

Typically, patients with CRSDs display chronic symptoms of insomnia too^12,14^. Evidence linking insomnia to markers of circadian dysfunction, such as late or advanced body temperature and cortisol rhythms or increased mean body temperature at night in different insomnia phenomena, show dysregulation of this process^14^. As mentioned before, older adults are more likely to develop chronic insomnia due to changes in homeostatic sleep drive and circadian rhythm. It has been suggested that older people often present an advanced sleep phase (falling asleep early and waking up early). However, these physiologic changes seen with increasing age are not always true for older people with insomnia symptoms. When compared with healthy individuals, these subjects tend to have a higher delayed circadian phase (circadian dispersion and lack of synchronization) and early awakenings, which extend insomnia problems during the night^13,14,24^.

### Shift work: a model of circadian misalignment

A social condition that mimics an extrinsic circadian rhythm disturbance or misalignment is the shift work, because of disruptions of the biological processes that regulate sleep and wake^57,58^. Usually, shift workers report reduced subjective sleep quality, as well as total sleep time^18^, which quickly result in a significant slept debt.

Shift work takes place on a working schedule outside the classical 9 am - 5 pm and it can be classified in one of two ways: rotating shift work (early morning, evening or night shifts) or permanent shifts (constant work pattern that may occupy unusual hours of the day)^42,56,57^, which contrasts with a more standard pattern^59^. Thousands of people have jobs demanding shift schedules, which is an important component of the contemporary economy due to the needs of companies and governments in providing around-the-clock services and products. Shift work is affecting 20% to 25% of employees and is becoming increasingly prevalent in contemporary life all over the industrialized world^43,57,59-62^.

Immediate symptoms associated with shift work, often short-term or related to specific phases of the work schedule, are sleep disturbances, sleep loss and fatigue. However, the symptoms can sometimes reflect a more serious and chronic disease process, such as impaired mental health, deficits in the cognitive domain^57^, and cardiovascular and associated-metabolic events^36,40,41,43,58,59,61,63-69^. For instance, there is a potential higher risk of cancer among shift workers, owing to reduced melatonin secretion, although this association is still a bit speculative^70^.

In connection with the point previously mentioned, in recent years several studies have examined associations between shift work and cardiometabolic risk factors, as we will demonstrate later.

### Shift work disorder

Despite most shift workers experience circadian disruption and sleep curtailment, not all have the circadian rhythm shift work sleep disorder (SWD) which is characterized by functional impairments that are associated with insomnia and/or excessive sleepiness during wakefulness^57,61^. It is estimated that 10% of the night and rotating shift workers and 1% of the population meet criteria for SWD^71^, having a shorter sleep duration, worse sleep quality and poorer performance on memory tasks, greater prevalence of gastric ulcers and depressive symptoms, and a greater incidence of risk factors than shift workers without SWD^59,62,71^.

However, Booker et al.^67^ suggested that the relationship between mental health and SWD is not well described until the date.

### Circadian misalignment and cardiometabolic risk

As most physiologic systems have a circadian component, shift workers often experience a cascade of biological consequences that leads to putative effects on physiological homeostasis. These are related to inflammation, oxidative stress, changes in patterns or levels of several hormones, reductions in physical activity, and poor dietary habits^18^. The pathways involved include rhythm disruption, lifestyle changes, job strain and social stress^60^, and this complex interaction of biopsychosocial factors predisposes individuals to an increased health risk^18^.

Given that shift workers stay awake and eat during the circadian phase that is appropriate for sleep and fast; and try to sleep during the time suited for activity and food intake (inversion of the human activity-rest cycle), the physiology and metabolism of those workers are compromised. Shift workers are exposed to abnormal light-dark cycles, which can suppress the production of melatonin and subsequently influence heart rate, cortisol and temperature during the biological night^18,72^.

There is considerable epidemiological evidence about the adverse downstream effects of shift work on cardiometabolic regulation^42^, because of chronic circadian misalignment and eating abnormal circadian times^52^. The increased risk of metabolic syndrome among shift workers has been less documented than cardiovascular diseases^19^. Furthermore, an asynchrony of the endogenous circadian rhythms, short sleep, and reduced melatonin levels also contribute to the development of other diseases or exacerbate existing disease, such as gastrointestinal musculoskeletal, neurological and reproductive disorders, besides an increased risk of developing heart attacks, sexual dysfunction and depression^4,18,20,43,53,56,57,73^.

### Shift work and risk factors

There are several risk factors for cardiovascular disease and metabolic syndrome that we must pay attention to. When compared with day workers, shift workers are more likely to develop insulin resistance in the liver^54^, impaired endothelial function, larger BMIs and to have higher levels of either total cholesterol or triglycerides and lower levels of high-density lipoprotein (HDL)-cholesterol^20,21,51,74,75^. However, the results of the studies conducted from 2001 to 2011 focused on the impact of shift work on the last parameter (HDL-C) are mixed: not all of them agree that shift work influences this parameter^59^. An association often appears when considering a certain age range (younger than 50 years)^76^ or taking into account confounders (e.g., socioeconomic and work- and lifestyle-related factors)^19^ or long duration of exposure (20 years)^19,59^. Beyond these established risk factors, alterations in markers of glucose and lipid metabolism, including hyperglycemia and dyslipidemia, respectively, are also present, contributing to metabolic abnormalities that increase the risk of CVD, obesity and diabetes^54^, as we can observe in Figure 3. However, nigh shift workers have a higher risk of diabetes, blood pressure, breast cancer and heart disease^75^. Regarding hypertension, circadian misalignment contributes to increased rates in shift workers and higher levels of blood pressure^20,42,52^, depending on age and duration of exposure^59^. Moreover, circadian misalignment decreases wake time cardiac vagal modulation, where the vagal parasympathetic activity is typically considered being cardioprotective^74^.

Some authors showed controversial results and concluded about a causal link between circadian misalignment/shift work and cardiometabolic risk^58,60,69^. The review published in 2015 by Gan et al.^69^ demonstrated that 18 of 28 independent reports showed a negative association between shift work and diabetes mellitus. Similarly, the research findings of Hulsegge et al.^65^ suggested that shift work was not related to an increased risk of cardiometabolic risk factors, except for overweight/BMI. In another study, Stenvers et al.^54^ stated that although several animal studies show that shift work causes increased food intake, increased body weight and disturbed glucose metabolism, the chronic effects of shift work have not been studied experimentally in humans. Nevertheless, several recent studies with humans have shown that the energy intake of night workers is not higher than day workers. These studies have mainly discussed the timing of these meals and their composition. And it is these factors that increase the cardiometabolic risk. When an adjustment for confounding factors such as age and BMI were performed, shift workers reported a greater energy consumption than day workers^75,77^.

In summary, it is known that several markers of cardiac function and metabolism display an endogenous circadian rhythm independently of behavioral and environmental changes. During shift work, pathophysiologic changes occur, leading to disturbances of circadian clock functioning and therefore, to circadian misalignment. This misalignment seems to increase the incidence of CVD and metabolic syndrome. Although the increased risk of shift workers with greater circadian misalignment has been hypothesized, the exact contribution of each risk factor and mechanisms involved need to be studied^18,72^.

### Impact of insomnia disorder and circadian misalignment on cardiac autonomic function in humans

Given the literature indicating that autonomic control and sleep regulation are interconnected through shared physiological, neurochemical and anatomical pathways^78^, one possible pathophysiological mechanism that may explain the adverse effects of insomnia and circadian misalignment is alterations of the autonomic nervous system (ANS), with a global sympathetic overactivity and/or parasympathetic suppression^4,79^. This sympathovagal imbalance contributes to elevated heart rate, blood pressure (strongly influenced by the transition across sleep stages^78^), promotes the formation of artery-clogging deposits, inhibits pancreatic β-cell function and insulin secretion, and is associated with immune dysfunction and inflammation; all of which have been associated with cardiometabolic morbidity and death^34^.

Heart rate (HR) controlled by both the sympathetic and parasympathetic nervous system and determined by the circadian system, and heart rate variability (HRV), mostly influenced by the parasympathetic nervous system, are measures of cardiac autonomic activity and markers of cardiovascular disease and mortality. Both variables provide information about the functioning of the branches of the ANS^25,80,81^. A higher HR and lower HRV are associated with CVD risk and an elevated HRV represents a healthy cardiovascular autonomic function^34^. In healthy subjects, the highest vagal influence on HR occurs during non-REM sleep (non-rapid eye movement), together with the highest feedback contribution of the baroreflex, consistent with a cardiorestorative role of non-REM sleep^80^. In contrast, REM (rapid eye movement) sleep is characterized by a marked sympathetic activation associated with blood pressure and heart rate instability, which supports the observation of increased prevalence of cardiovascular events in the early morning^78^.

Since shift work schedules produce misalignment between the endogenous circadian rhythmicity and the timing of sleep and wakefulness; and HR and HRV are dynamically influenced by that, it is important to understand the impact of the misalignment on those variables. For instance, Grimaldi et al.^82^ determined the impact of circadian misalignment on autonomic nervous system control of cardiovascular function, suggesting that shift workers might have a reduction of cardiac vagal modulation during sleep and an increased risk of developing adverse cardiac events. Wakefulness is additionally common in shift workers due to the activation of the nuclei of the ascending arousal system caused by projections from areas of the hypothalamus^80^.

**Figure 4:**
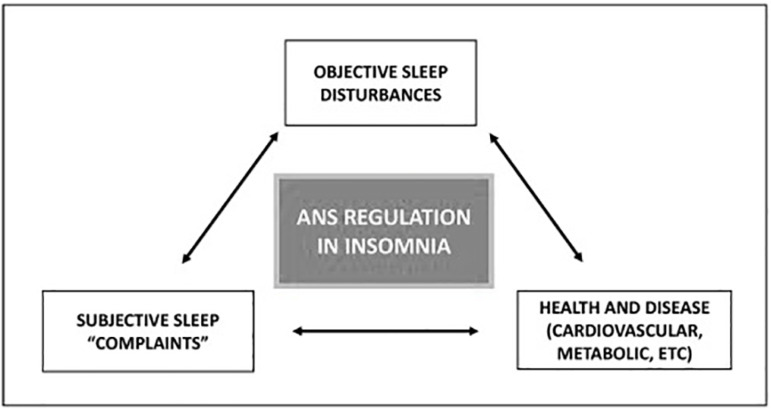
Schematic representation of the central role played by the autonomic nervous system (ANS) in mediating the complex interaction between subjective and objective sleep disturbances and health outcomes in insomnia. (Adapted from Grimaldi et al.^78^.)

Furthermore, insomnia with objective short sleep duration has also been associated with cardiovascular autonomic dysfunction (see Figure 4), leading to an increased HR, decreased HRV^25^ and physiological hyperarousal (e.g., hyperactivity of the HPA axis, increased daytime MSLT (Multiple Sleep Latency Test) sleep latency, sympathetic activation and anxiety about sleep)^25,78^. Increased sympathetic activity is also associated with higher levels of plasma urine norepinephrine in both short sleepers and insomniacs^4^. However, while evidence suggests low HRV is associated with more severe sleep disturbances, within the context of insomnia, findings vary across studies^34,78^. Jarrin et al.^34^ showed that there are little data on whether cardiovascular function differs between patients with different insomnia phenotypes and the study of Vgontzas et al.^25^ failed to confirm previous findings of an increase in sympathovagal balance and a decrease in parasympathetic nocturnal activity. A recently published review from Grimaldi et al.^78^ reports that although alterations in ANS activity have gained progressive attention as a pathophysiological link between insomnia and cardiometabolic risk, the specific mechanisms involved remain unknown. Hence, they proposed the hyperarousal hypothesis to help explain the relationship between insomnia and ANS activity. The results from the literature to support this hypothesis are inconclusive, but some of them include findings that individuals with insomnia have heightened indices of cortical activation (e.g., EEG (electroencephalogram) beta activity during sleep), peripheral and central ANS activation (e.g., increased nocturnal cortisol, core body temperature, heart rate, and norepinephrine) and psychological hyperarousal.

## CONCLUSION

A critical review of the literature provides some evidence that cardiovascular autonomic dysfunction may have a significant contribution to the cardiometabolic risk associated to inadequate sleep/insomnia, with several studies showing that both acute and chronic insomnia are associated with adverse cardiometabolic outcomes, such as hypertension, diabetes, increased inflammation, impaired glucose tolerance, CV disease, and neurological or psychiatric issues. Some of those studies reported that a mismatch of circadian rhythms triggers a cascade of negative consequences in several biological processes, compromising the human homeostatic systems and the dual component of sleep regulation. Autonomic cardiovascular dysregulation is a plausible mediator of this negative impact that deserve further research.
